# Nanoparticle-Based Drug Delivery Systems for Photodynamic Therapy of Metastatic Melanoma: A Review

**DOI:** 10.3390/ijms222212549

**Published:** 2021-11-21

**Authors:** Nkune Williams Nkune, Heidi Abrahamse

**Affiliations:** Laser Research Centre, Faculty of Health Sciences, University of Johannesburg, P.O. Box 17011, Doornfontein 2028, South Africa; nkune.williams@gmail.com

**Keywords:** metastatic melanoma, photodynamic therapy, passive or active targeted delivery, PS nanoparticle-mediated platforms, three-dimensional (3-D) cell cultures

## Abstract

Metastatic melanoma (MM) is a skin malignancy arising from melanocytes, the incidence of which has been rising in recent years. It poses therapeutic challenges due to its resistance to chemotherapeutic drugs and radiation therapy. Photodynamic therapy (PDT) is an alternative non-invasive modality that requires a photosensitizer (PS), specific wavelength of light, and molecular oxygen. Several studies using conventional PSs have highlighted the need for improved PSs for PDT applications to achieve desired therapeutic outcomes. The incorporation of nanoparticles (NPs) and targeting moieties in PDT have appeared as a promising strategy to circumvent various drawbacks associated with non-specific toxicity, poor water solubility, and low bioavailability of the PSs at targeted tissues. Currently, most studies investigating new developments rely on two-dimensional (2-D) monocultures, which fail to accurately mimic tissue complexity. Therefore, three-dimensional (3-D) cell cultures are ideal models to resemble tumor tissue in terms of architectural and functional properties. This review examines various PS drugs, as well as passive and active targeted PS nanoparticle-mediated platforms for PDT treatment of MM on 2-D and 3-D models. The overall findings of this review concluded that very few PDT studies have been conducted within 3-D models using active PS nanoparticle-mediated platforms, and so require further investigation.

## 1. Introduction

Cancer refers to a variety of diseases caused by erratic proliferation of malignant cells, which can invade other parts of the body distant from the site of origin [1]. According to the statistics reported by the World Health Organization (WHO), over one million new cancer cases are reported annually, which is predicted to reach 13.1 million by the year 2030 [1]. Skin cancer is one of the commonly diagnosed malignancies and its incidence has risen rapidly in recent years [2]. There are three types of skin cancers: basal cell carcinoma (BCC), squamous cell carcinoma (SCC), and melanoma. BCC and SCC are non-melanoma skin cancer (NMSC), since they do not originate from skin melanocytes and are relatively non-invasive [3]. However, cutaneous malignant melanoma is traditionally considered to be metastatically invasive due to its ability to invade and spread to neighboring tissues [4].

Melanoma is the most grim skin malignancy triggered by both intrinsic and extrinsic factors [5]. This form of cancer emerges from melanocytes, which are the cells located in the deeper layers of the epidermis and produce melanin pigments [6]. Thus, it is caused by a deformed single melanocyte or dysfunction of dysplastic nevi [6]. Cutaneous melanoma is the most prevalent form, accountable for almost 90–95% of all melanoma cases [7], which tends to predominately spread to the brain, eyes, anus, liver, and bone [7]. Melanoma is staged based on their degree of involvement and dissemination to lymph nodes and other surrounding healthy tissues [8]. Stage I and stage II neither exhibit any lymph node involvement nor metastasis whereas stage III melanoma shows local lymph node metastases [8]. Metastatic melanoma (MM) is considered as an advanced phase of stage IV skin cancer characterized by the metastasization of malignant cells from the site of origin to distant organs of the body [4]. Therefore, early detection of this cancer is vital for efficient therapy because late stages are non-curable, resulting in a high mortality rate [9].

The most common risks associated with melanoma include intensive exposure of the skin to ultraviolet radiation (UV), age, gender, immunodeficiency, and family history [10]. Skin pigmentation plays a pivotal role in influencing MM morbidity and mortality rates [10]. Furthermore, light-skinned people lacking melanin are more susceptible to UV radiation-induced DNA destruction compared to their dark-skinned counterparts [4]. The rapid rise in melanoma cancer cases has not been mitigated by the discovery of improved treatment approaches in recent decades [5]. Hence, the alarming upsurge in MM-related morbidity and mortality remains a major challenge in healthcare globally [11].

MM treatment based on location, stage, and genomics incudes surgery, chemotherapy, radiotherapy, immunotherapy, and molecularly targeted therapy [9]. However, these treatments often cause undesirable side effects [9]. Surgery is the mainstay treatment for early stage melanoma to circumvent metastasization and enhance survival rates [12] while radiotherapy is highly recommended for the treatment of bone, skin, and brain metastases [9]. For decades, chemotherapeutic drugs, such as dacarbazine (DTIC), temozolomide (TMZ), and fotemustine, have been effective treatments for MM [8]. Although, they can cause undesirable adverse effects on surrounding normal cells [13], chemotherapy remains indispensable in the palliative therapy of resilient, progressive, and relapsed tumors [9].

Amongst immunotherapies, neutralizing antibodies with a high targeting affinity for immune blockades, such as cytotoxic T lymphocyte-associated protein 4 (CTLA-4) and programmed cell death 1(PD-1), have enhanced patients’ survival prospects [7]. Furthermore, treatments with target specificity for the oncogenic serine/threonine-protein kinase B-Raf (BRAF) proteins, which are universally expressed in melanoma cases, have also shown a significant effect against MM [7]. However, these therapies are also hampered by drug resistance and adverse immunological reactions in patients [13].

The largest proportion of patients who have initial and considerable tumor relapse, may experience disease progression within 8 months post-treatment [7]. Therefore, there is a dire need for more effective therapies to overcome drug resistance and expand the options available for MM patients [7]. Among the various cancer treatment modalities, photodynamic therapy (PDT) has emerged in recent years as an ideal treatment to circumvent challenges faced by melanoma treatments [5]. PDT is based on the uptake of photosensitizers (PSs) by tumor cells, followed by their excitation using a suitable wavelength of light, which induces tumor damage due to the generation of cytotoxic reactive oxygen species (ROS) [4]. PDT has several advantages over conventional anticancer therapies, including a reduced invasiveness compared to surgery, precise tumor targeting ability, low morbidity, and desired patient tolerability [4].

Most cell-based experiments typically use conventional two-dimensional (2-D) monolayer cell cultures, which poorly resemble the three-dimensional (3-D) cellular environment in the human body, such as cellular heterogeneity, poor distribution of oxygen and nutrients, growth kinetics, cell to cell interactions, and the extracellular matrix (ECM) [14]. Two-dimensional monolayer cell cultures present several advantages, such as easy cell preparation, maintenance, and manipulation [14,15]. However, the growth of cells on a flat surface does not accurately integrate essential interactions between the cells and the adjacent ECM observed in vivo, which mainly consist of type 1 collagen fibril structural protein [16]. Furthermore, cell-to-cell interactions are limited in 2-D models because the primary interaction is with the host’s plastic surface [16]. The lack of cellular interactions in 2-D models may cause the adhesion properties and architecture of cancer cells to differ from their in vivo counterparts, thereby affecting cellular proliferation and signaling mechanisms, as well as cellular responses to therapies [17,18]. Recently, rapid developments in nanotechnology applications for cancer therapy have been promising [13]. Nanoparticles (NPs) can directly eliminate cancer cells or can serve as carriers for chemotherapeutic drugs, PSs, and gene therapy [13]. In addition, NPs can accumulate passively or actively in cancers to improve treatment specificity, while mitigating undesirable side effects [4,13].

Xu et al. [17] reported that when NPs and drugs are administered to monolayer cell cultures, they are able to penetrate into cells without being subjected to any physical limitation, whereas NPs delivered in vivo encounter obstruction by the ECM surrounding tumor cells [17]. Thus, the exposure of cancer cells in monolayer culture to a uniform setting with a steady supply of oxygen and nutrients prevents them from simulating in vivo cancer tissues, resulting in altered gene expression patterns [15]. In solid human tumors, cancer cells grow and proliferate by integrating with the surrounding connective tissue, known as stroma, and the ECM is the most important component of in vivo connective tissues [15]. The 3-D cell cultures integrating ECM materials, such as collagen, can serve as better cancer models in cancer research for the evaluation and testing of novel drugs since they resemble the fundamental aspects of the in vivo human cellular environment [19]. Techniques, such as scaffolds, hanging drops, ultra-low attachment plates, micropatterned plates, spinner flasks, and microfluidic devices, are widely used for generating in vitro 3-D cell culture models [20]. The aim of this review is to investigate active targeted PS nanoparticle-mediated delivery platforms that have been used for treatment of 2-D and 3-D tumor spheroids of MM.

## 2. Photodynamic Therapy

**Figure 1:**
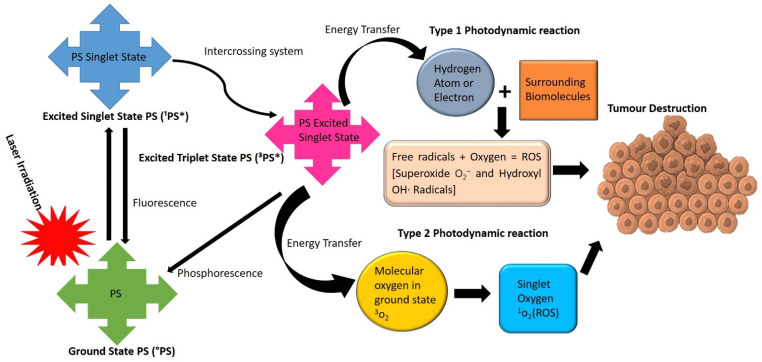
Type I and type II photodynamic reactions following illumination of a PS with an appropriate wavelength of laser light. ^1^P* = Excited Singlet State PS and the ^3^P* = Excited Triplet State of the PS.

PDT is a novel phototherapeutic modality for oncological diseases [21]. It incorporates three fundamentals to induce cellular damage: a photosensitizer (PS), surrounding tissue molecular oxygen, and visible light coinciding with the absorption spectrum of the PS [21]. PDT can trigger photochemical reactions to obliterate localized tumor, upon photoactivation of photosensitizing agents (PSs) [22]. PDT induces cell death via two main oxygen-dependent mechanisms, namely type I and type II photodynamic processes, which are shown in Figure 1. In type I photochemical reactions, the excited triplet state of the PS transfers its energy to the surrounding biomolecules [23]. An interchange of either a hydrogen atom or an electron occurs between the PS and the tumor tissue (substrate), which results in the formation of free radicals [24]. The generated free radicals then interreact with oxygen molecules, which further creates reactive oxygen species (ROS), such as superoxide and hydroxyl radicals [24].

In type II photodynamic reactions, the energy is directly transmitted between the excited PS and the basic ground state of oxygen, which in turn generates a remarkably oxidizing singlet oxygen [25]. The generated ROS and singlet oxygen species can denature proteins and disrupt lipids and other organelles within the tumor site, resulting in either apoptotic, necrotic, or autophagy cell death pathways [25].

### 2.1. PDT-Mediated Modes of Cell Death Induction

The photo-activation of administered photosensitizing agents with an appropriate wavelength of light initiates the PDT-induced cancer cell death mechanisms [26]. Apoptosis, necrosis and autophagy singularly or concurrently are the main PDT cell death pathways [27]. The degree of photodamage is determined by a number of variables, including the PS aggregation site, bioavailability, PS physicochemical properties, tissue molecular oxygen concentration, and wavelength of light and intensity [4]. The mitochondrial damage can induce apoptosis [23], which is essentially the most predominant mechanism evoked by PDT effects, via diverse mechanisms in combination with caspases, Bcl-2 proteins, and proapoptotic factors [27]. Additionally, cell membrane damage and loss of integrity lead to necrosis, while the damage to the lysosomes or endoplasmic reticulum can evoke autophagy [23]. However, when ATP levels drop in the treated cells, PDT tends to trigger autophagic and necrotic modes of cell death induction [27]. Autophagy is an intricate programmed cell death that rejuvenates cells through conservation of their nutrients and degradation of intracellular protein aggregates and impaired organelles [28]. This form of programmed cell death serves as a tumor suppressor and promoter, hence it is not ideal in cancer therapy as cells can recover and cause tumor recurrence [29].

Necrosis is an uncontrolled cellular destruction induction that obliterates an extensive population of cells [27]. It is differentiated by cytoplasmic expansion, drastic annihilation of organelles, and plasma membrane disruption, which triggers the release of intracellular contents and inflammation [30]. Studies by van Straten et al. [31] showed that cancer cells subjected to an intense PDT dose (PS concentration and light intensity) could drastically undergo necrosis rather than apoptotic cell death [31]. Furthermore, they noted that light-activated photofrin PS exhibited an inhibitory effect via apoptosis when it aggregated in the cytoplasm, lysosomes, mitochondria, and Golgi apparatus [27], whereas it triggered necrosis due to high accumulation in the cellular plasma membranes and nuclei of the target tumor cells [31].

### 2.2. PSs Utilized in Metastatic Melanoma Treatment

PSs are photoactive molecules, which are activated with an appropriate wavelength of light to trigger photochemical and photophysical reactions [23]. PSs are generally classified as first, second, and third based on historical development and their distinct properties [31]. Ideally, a PS is characterized by its high tumor affinity, negligible cytotoxicity in the dark, strong light absorption in the range of 600–800 nm, high chemical purity and stability, high quantum yield of ROS generation, rapid body clearance, and ease of synthesis [22,23].

The application of first-generation PSs, haematoporphyrin derivative (HpD) and photofrin, was hampered by their poor chemical purity, low affinity for targeted cells, skin hypersensitivity, and poor tissue permeation due to their maximal absorption at relatively short wavelengths (>650 nm), as well as their delayed clearance from the body [23,27]. Thus, second-generation PSs were introduced to circumvent the shortcomings of the first-generation PSs [31]. The second-generation PSs are synthetic chemicals composed of porphyrins, chlorins, phthalocyanines, benzoporphyrin, bacteriochlorin, curcumin, methylene blue analogues, and many more [22]. They exhibit a higher degree of chemical purity than first-generation PSs, enhanced singlet oxygen quantum yields, due to their longer absorption wavelengths, together with improved penetration to deep-seated tumors [21]. In addition, they demonstrate negligible side effects, which is attributed to increased tumor selectivity and rapid elimination of the PS from the body [23]. Among the second-generation PSs, phthalocyanines (Pcs) are widely used PSs containing a central diamagnetic metal ion (e.g., zinc, aluminum, or magnesium) that significantly increases the triplet-state quantum yield of ROS generation and photostability, as well as achieving deeper tissue penetration with minimal side effects [32]. Studies by Valli and colleagues [33] noted that photoactivated zinc phthalocyanine (ZnPcS) demonstrated an increased level of ROS generation, which triggered apoptosis and necrosis in MM cells [33].

**Figure 2:**
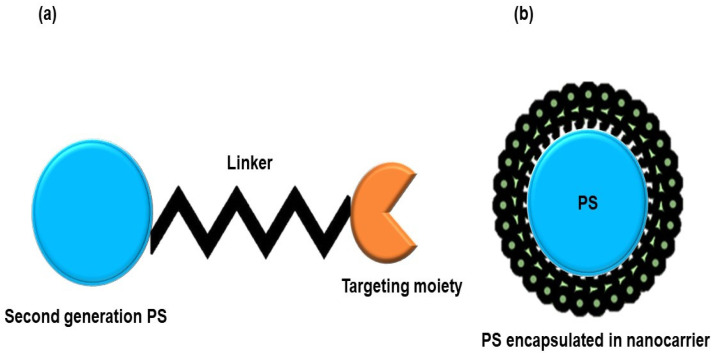
Illustration of third-generation PSs: (**a**) Second-generation PS functionalized with a targeting biomolecule. (**b**) Second-generation PS in combination with a nanocarrier.

Table 1 reveals several PSs that have been explored in the PDT treatment of MM. The overall findings from Table 1 conclude that second-generation PSs are potent photoactive agents for PDT treatment of MM cancer. However, the major drawback of second-generation PSs is their inherent hydrophobicity, which drastically limits their clinical application and necessitates the search for novel drug delivery approaches [23]. To address this issue, third-generation PSs have emerged [31]. The third-generation PSs are second-generation PSs that have been incorporated with targeting entities, such as antibodies, peptides, and carbohydrates, or encapsulated into biological carriers, for example, nanoparticles, liposomes, and micelles (Figure 2) [22]. Therefore, the bioavailability and targeted specificity of PSs are improved in cancer cells while leaving surrounding normal tissues unaffected, due to the high affinity of targeting moieties to cancer cell surface antigens compared to normal cells [22].

### 2.3. Limitations of PDT in Metastatic Melanoma Treatment

As previously highlighted, PS light activation in the availability of molecular oxygen is an indispensable process in PDT [11]. Preferably, light applied in PDT must be strongly absorbed by the administered PS while being absorbed negligibly by surrounding biological entities [5]. Biological tissues typically demonstrate poor absorption of light within the optical window (650–800 nm) [5]. In the case of MM, however, the scenery is different due to the presence of high levels of melanin [51]. It has been stated that melanin promotes the resistance of MM towards PDT, by acting as an optical shield obstructing light from reaching the targeted site [51]. Melanin predominately absorbs light at the critical PDT therapeutic window (500–600), while the transmittance in melanotic melanomas only takes place beyond 700 nm [5,51]. Hence, melanin is considered as a major competitor of PS for light absorption in PDT [5]. Furthermore, studies showed that melanin acts as an antioxidant and an ROS scavenger, conferring melanoma resistance to PDT [11]. In order to study the influence of melanin on PDT outcome, studies compared the susceptibility of pigmented (e.g., B16F1 and B16F10) and less pigmented cell lines (such as A375) to PDT [52,53]. These studies corroborated that the cells with low melanin levels were more prone to PDT-induced cell death [5]. Therefore, a PS with the ability to impede melanogenesis or trigger depigmentation would be a valuable addition to the therapeutic modalities for treating MM more effectively [11].

Solid tumors, such as MM, are characterized by heterogeneous cell proliferation, which causes oxygen scarcity, causing a decreased in the blood supply and hypoxic tumor microenvironment [54]. The hypoxic tumor microenvironment is responsible for PDT treatment failures and also promotes cell growth, invasion, and metastasis of tumors [55,56]. In this regard, the incorporation of nanotechnology and cellular biology may open up new avenues for combating hypoxia and considerably enhance PDT outcomes [56].

## 3. Nanotechnology

Nanotechnology is a multidisciplinary field that aims to revolutionize cancer detection and therapy by designing biological materials, such as molecules, atoms, and supramolecules, at a nanometer range (1–100 nm) [57]. Nanoparticles (NPs) provide unique properties, such as permeability, hydrophilicity, stability, porosity, and large surface area to volume ratio [1]. These properties enable NPs to facilitate drug delivery and small compounds into cancer cells, and improve the intra-tumor drug concentration with negligible effects on healthy tissues [58]. NPs are ideal for intravenous drug administration because they can localize very selectively in cancerous cells via an enhanced permeability and retention effect (EPR) [59]. The EPR phenomenon is related to the dysfunctional lymphatic networks within the tumorous site, which allows drugs encapsulated in NPs to easily pass through leaky tumor vasculature [59]. The EPR is influenced by various factors, such as the pore dimensions of the administered molecule to the tumor and the tumor site. Therefore it is of great importance to optimize the size of NPs to enhance drug delivery [4].

Novel nanosystems are generally categorized into organic and inorganic nanocarriers (Table 2). Their physiochemical characteristics can be optimized by modifying their size, chemical composition, morphology, and surface properties to design a smart drug delivery system [60]. Surface modifications of NPs with polyethylene glycol (PEG) help the nanocarrier to evade biological barriers (e.g., macrophages) and consequently accumulate at target sites [58]. Another important approach is the bioconjugation of biomarkers onto the surface of NPs, which can enhance the target specificity to overexpress drug-loaded NPs at targeted regions with minimal accumulation in healthy tissues [4].

### 3.1. Application of Nanotechnology in PDT Treatment

The application of nanotechnology in PDT has paved new avenues for cancer treatment by offering precise PS delivery platforms to targeted regions with low toxicity to normal tissues [5]. In recent years, the incorporation of NPs with PSs has been under intense investigation to tackle the fundamental challenges encountered by classical PDT [23]. NPs can significantly improve PSs’ solubility in water, due to their inherent hydrophilicity and thereby enhancing their cellular uptake [63]. NPs protect conjugated PSs from unwanted degradation since they can bypass immune system barriers, allowing for a prolonged release of the PS [67]. Moreover, they can accommodate a large amount of anticancer drugs to cancer tissue due to their high surface area-to-volume ratio [68]. Small NPs can easily penetrate cancer cells due to the EPR effect [67]. Other advantages of NPs include their high biocompatibility, highly modifiable surface chemistry, and versatility in loading different drugs and targeting agents for multiple functions, which have made them an ideal candidate in PDT [4]. NPs have improved pharmacokinetic parameters of PDT, such as good clearance values, large volumes of distribution, and greater bioavailability in cancer cells via the EPR effect [69]. Thus, NP-based drug delivery systems in PDT are fast becoming popular.

### 3.2. Passive PDT Nanoparticle-Mediated PS Delivery Platforms for MM Treatment

Nanocarrier systems in PDT hold great promise for improved PS absorption in malignant cells due to enhanced PS cargo stability, allowing tumor targeting, minimizing the off-target effects of the PS cargo, and coordinating the release kinetics of the PS cargo [12]. Large surface to volume ratios of NPs facilitate carrying of large amounts of PSs with different physical chemistries, thus improving the PS delivery concentration and retention either passively or actively at target tissues [12,68]. Furthermore, in vivo circulation and passive tumoral uptake of the PSs is enhanced as drug-loaded NPs can simulate biological matter [68]. This is attributed to the ability of the NP-mediated PS delivery platforms to evade immune system checkpoints, allowing for improved bioavailability and PS localization in diseased tissues [70]. Extensive studies have been conducted into effective PS delivery platforms using nano-drug carriers for PDT treatment of MM, which are summarized in Table 3. However, since passive PS nanocarrier systems cannot exclusively discriminate cancer cells from healthy cells and thus sometimes accumulate in normal tissues, researchers have been actively engaged in developing active targeting nanocarrier systems with biomolecules to specifically target receptors overexpressed by cancer cells only [27].

### 3.3. Active PDT Nanoparticle-Mediated PS Delivery Platforms for MM Treatment

**Figure 3:**
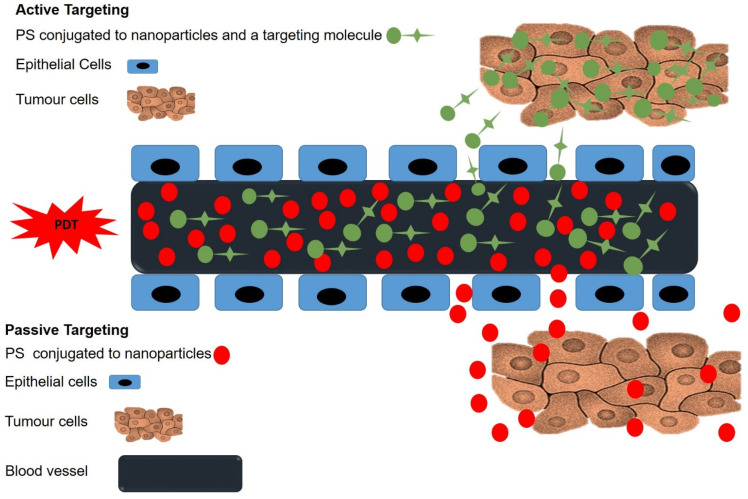
Passive and active targeting mechanisms in PDT. Passive uptake of PSs is facilitated by the EPR effect, while active uptake of PSs involves targeting moieties, which have specific affinity for tumor cell antigens.

To improve PSs’ cellular uptake and localization in MM cells, active PS nanocarrier systems have been developed [86]. These involve the incorporation of PS nanocarrier systems with active targeting entities (i.e., antibodies, aptamers, peptides, folic acid, carbohydrates, DNA/RNA, and antibody fragments), which have a high binding affinity for receptors overexpressed by MM cells, allowing direct PS delivery to the target site (Figure 3) [21,86].

**Figure 4:**
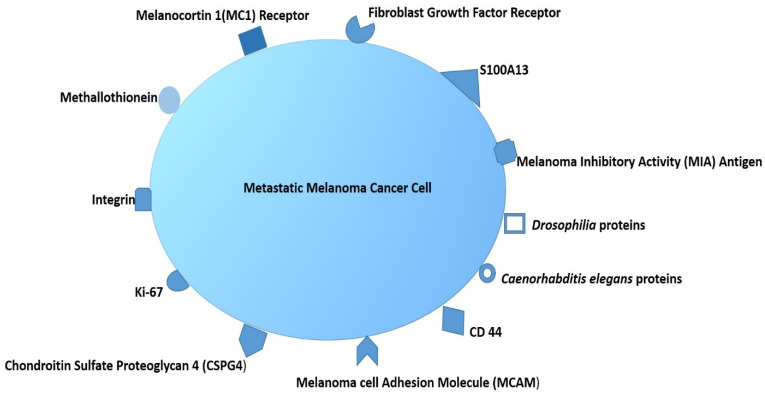
An overview of common antigens expressed by melanoma cells.

Monoclonal antibodies (mAb) are the most recommended targeting agents for cancer cell antigens in order to improve the specific and active targeted PS nanocarrier systems and so enhance the overall efficacy of PDT [68]. Research has highlighted that MM tumor cells typically overexpress surface proteins, such as melanoma inhibitory activity (MIA), TRAIL-receptor, B cell lymphoma 2, integrin α and β proteins, mitochondrial p32 protein, extracellular matrix 1, and a fusion of *Drosophilia* protein, *Caenorhabditis elegans* protein, and ephrin type-A receptor 2 (Figure 4) [4,86,87,88,89]. Currently, trastuzumab, rituximab, and bevacizumab are FDA-approved mAbs that can be utilized for targeting MM cells [86]. In recent years, great strides have been made in terms of receptor-specific targeting PS encapsulated in nanocarriers, which are further modified with mAbs, to improve PS intracellular accumulation in targeted cells only [90]. However, mAb-mediated active drug nanocarrier applications are very costly, and larger-scale production is hampered by their physical and chemical properties, which necessitate rigorous characterizations to ensure the structural composition is unaffected during manufacturing to mitigate undesirable side effects [4].

It should also be kept in mind that virtually all the published data regarding cancer biology were obtained from conventional monolayer cultures, and their functional properties do not resemble human tumor, and in vivo clinical studies [20]. Studies by Naidoo et al. (2019) investigated a novel active ZnPcS_4_ nanobioconjugate, for the PDT treatment of two-dimensional (2-D) in vitro MM A375 cells. The results suggested that the 2-D monolayer cell cultures do not fully translate what happened in the clinically setting and so the three-dimensional (3-D) tumor spheroid cell culture was of great importance to such investigation [91]. With reference to Table 4, a number of studies in relation to active-mediated NP delivery platforms for the treatment of MM have been conducted on 2-D monolayer and animal studies. Thus, more research is needed within 3-D cell culture models.

### 3.4. Applications of Active-Mediated NP Delivery Platforms in PDT Treatment of 3-D Tumor Models of MM

In order to bridge the gap between 2-D monolayer cell cultures and in vivo tumor models, it remains imperative to evaluate the therapeutic efficacy of PDT using 3-D platforms that can readily recapitulate human response [14,99]. Since conventional 2-D monolayer static cell cultures fail to mimic inherent 3-D tissue structure, significant discrepancy has been noted when transferring results from 2-D culture to in vivo tumor tissue models [20]. Therefore, animals studies based on murine models remain tremendously useful and are considered to be the most common strategies for screening and testing novel drugs [20,99]. However, these models are very expensive and time-consuming [20]. In addition, murine models essentially contain non-human host cells, and thus they still do not fully mimic the pathological or physiological mechanisms in humans [100,101]. In this sense, 3-D tumor models can serve as ideal platforms to resemble different aspects of human tumors and evaluate the efficacy of active nanoparticle-mediated PS delivery systems [100,101].

Studies by Yuan et al. [102] investigated the phototoxicity of Chlorin e6 (Ce6) conjugated to PAMAM dendrimer (generation 7.0) functionalized with RGD peptide, to enhance PS cellular uptake and tumor penetration in A375 tumor spheroids. The constructed targeted nanobioconjugate (RGD-P-Ce6) was uniform and monodispersed with a diameter of 28 nm. A375 spheroids were incubated with free Ce6 and RGD-P-Ce6 (800 nM) and then irradiated with a 660-nm laser at 6.3 J/cm^2^. RGD-P-Ce6 resulted in a significant 25.7% of early apoptotic cells, and 25.2% of dead cells 12 h post irradiation. It was also reported that the targeted nanobioconjugate showed 79.3-fold higher cellular uptake than free Ce6 [102]. The study concluded that the targeted nanobioconjugate improved cellular internalization via receptor-mediated endocytosis, which generated adequate singlet oxygen to induce cell death [102]. Tham et al. [103] developed a mesoporous nanocarrier loaded with phthalocyanine (Pc), dabrafenib, and trametinib to enhance their cellular uptake and tumor penetration in 3-D tumor spheroids and in vivo tumor models. The dual nanocomposite (PcNP-drug) was monodispersed with a hydrodynamic diameter of 78 nm with no aggregation. The study noted that the nanocomposite showed a far more improved cell-killing efficacy in spheroids than single treatments, with 8% cell viability [103]. In addition, PcNP-dug achieved 76% tumor regression and successfully targeted BRAF-positive cancer cells in vivo, while sparing non-BRAF-expressing normal cells [103].

However, in relation to active targeted PS nanoparticle-mediated delivery platforms for treatment of 3-D tumor spheroids of MM, very few studies have been conducted and so require further investigation to potentially bridge the gap between preclinical and clinical studies.

## 4. Clinical Studies

Although the clinical application of PDT in MM treatment is still being debated, some clinical outcomes have been published. Barbazetto et al. [104] investigated the phototoxicity of verteporfin on four choroidal melanoma patients. The tumors were irradiated at a fluency of 100 J/cm^2^. The results noted that PDT triggered tumor regression in two cases while in the other cases, melanomas remained unresponsive and necessitated surgical excision [104]. Similarly, studies by Donaldson et al. [105] tested the effect of laser irradiation and verteporfin in a patient with choroidal amelanotic melanoma and noted a complete tumor regression [102]. The patient remained asymptomatic with no apparent tumor recurrence, 13 months after treatment [105].

A study by Soucek and Cihelkova [106] tested the antitumor effect of verteporfin with laser irradiation at a fluency of 100 J/cm^2^ on a 57-year-old male with subfoveal amelanotic choroidal melanoma, which indicated a drastic tumor regression [106]. In 2012, Tuncer and colleagues [107] investigated the combined effect of laser treatment and verteporfin on a 40-year-old female with amelanotic choroidal melanoma. Iodine brachytherapy noted no tumor regression at the 16-month check-up, whereas verteporfin-PDT significantly reduced tumor size by 5-fold, and the effect was still evident after 50 months of follow-up [107]. Similarly, Campbell et al. [108] evaluated the response of amelanotic choroidal melanomas to verteporfin-PDT in nine patients. The therapy triggered a complete tumor regression with no sign of relapse in eight cases. However, only one patient presented with two local recurrences [108]. The treatment revealed no serious complication or negative effects on vision [108]. Most recently, a study by O’Day et al. [109] reported that verteporfin with PDT had initial tumor regression in 88% of patients with choroidal amelanotic melanoma post the initial dose of PDT. However, 44% of these patients experienced recurrence with a mean follow-up of 42 months. Moreover, 12% of the cases were unresponsive to the treatment [109].

Turkoglu et al. [110] also investigated the effectiveness of PDT on 12 cases of eye melanoma, 10 of which were amelanotic and the other two had a slightly pigmented appearance. The treatment substantially decreased small amelanotic choroidal tumors in 8 cases over an average of 5 years [110]. A corresponding study by Fabian et al. [111] noted that PDT effectively eradicated lightly pigmented posterior pole choroidal melanoma in 80% of patients post three therapy sessions within a 6-month follow-up [111]. The overall findings from these studies conclude that PDT may be an ideal therapeutic approach for choroid melanoma because it has no negative effects on vision.

On the other hand, studies by Sheleg et al. [112] tested the effect of PDT with chlorin e6 (Ce6) on pigmented metastatic melanoma tumors. A dose of 5 mg/kg PS was administered intravenously to 14 patients, and laser irradiation with a fluency of 80–120 J/cm^2^ was applied 1 and 24 h after administration. The results showed that all melanoma skin metastases regressed following double PDT exposure, with no recurrence. The treatment had no effect on renal or hepatic function [112].

Significant efforts have been made to investigate PDT in combination with biologic therapy. Allo and colleagues [113] reported that PDT combined with the BRAF inhibitor vemurafenib resulted in significant tumor regression in a patient diagnosed with metastatic melanoma [113]. In a study performed by Canal-Fontcuberta et al. [114], the combined effect of PDT and bevacizumab on choroidal melanoma reduced tumor vascularity, lowering the risk of bleeding during the biopsy [114].

Although classical PDT has shown a fair safety profile in clinical settings, undesirable side effects, such as local swelling and burning discomfort, have been reported [115,116]. Hence, third-generation PSs integrated with NPs have been designed to minimize unwanted complications while effectively obliterating tumors (Table 3 and Table 4), which will facilitate further clinical investigation.

## 5. Conclusions and Perspectives

Metastatic melanoma remains a major health concern globally, and incidence rates have been on the rise in recent years [11]. It is often diagnosed in its advanced stages, which translates to a poor prognosis, low survival, and tumor recurrence [86]. Current therapeutic approaches for MM suffer from some resistance and fatal side effects, and invasive surgical excision [4]. In view of this, actively targeted PDT is a popular approach for the treatment of MM due to its non-invasiveness and limited side effects [86]. Moreover, the incorporation of NPs and PSs to passively and actively increase their affinity for tumors with less damage to surrounding healthy tissues has been extensively researched in order to improve PDT treatment outcomes [21]. The unique advantages of NPs minimizing PS leaching with a high PS loading capacity play important roles in PDT. Furthermore, NPs can enhance PS passive uptake via the EPR effect, and allow for ease of functionalization with various ligands to promote active PS cellular uptake for the overall enhancement of PDT MM treatment [117,118]. More importantly, PS nanocarriers tend to mimic biological processes to evade various immune system barriers, allowing for effective PS delivery and cellular uptake in tumors [68]. Although, PS nanocarriers may distribute in healthy tissues and cause unwanted side effects [27], conjugation of PS nanocarriers with targeting moieties allows them to be actively absorbed by MM cells only, and enhances PS accumulation in targeted regions, while reducing its concentrations in normal tissues [5]. It is speculated that, in the near future, PDT based on nanotechnology systems may be a novel approach for treating MM globally [5]. However, more research is still needed to investigate the physical and pharmacokinetic properties, tolerability, and safety profiles of nanocarriers, so that a desirable accumulation and PS uptake can be achieved in the targeted regions. Currently, the majority of PDT experiments have been conducted on conventional 2-D monolayer cell cultures. Compelling evidence suggests that various preclinical drugs fail clinical trials, thereby delaying the discovery of effective therapeutics [20]. This is because 2-D cell cultures lack cell-to-cell interactions and an intrinsic tumor microenvironment that influences tumor growth and therapy response, which result in phenotypic discrepancies when compared to the real tumors. Furthermore, animal studies are very expensive and do not simulate human tumors due to the influence of non-human host cells. In this sense, 3-D cell cultures hold great promise for bridging the gap between preclinical and clinical studies since they are scientifically accurate and mimic many aspects of real human tumors. Thus, spheroids have the potential to improve clinical efficacy predictability and may minimize, if not replace, xenograft models to a large extent in the near future. Together with emerging nanotechnology-based drug delivery systems, 3-D in vitro models are expected to significantly reduce the cost of new drug discovery, thereby making anticancer therapies increasingly more accessible to the public.

The overall findings of this review conclude that very few PDT studies have been conducted within 3-D cell culture models using active PS nanoparticle-mediated platforms. Thus, further application of functionalized nanocarriers for targeted PDT MM treatment by conducting more 3-D in vitro studies with more effective theoretical and mathematical models is required to expedite preclinical phases and yield successful clinical trials.

## Figures and Tables

**Table 1:** Studies of various conventional PSs used in the PDT treatment of melanoma.

Generation	PS	Wavelength (nm)	Fluency (J/cm^2^)	Dose	Cell Line	Tumor Model	Outcome	Ref.
1st	Porfimer sodium	630	10 and 100	0.1–10 µg/mL	MCC ^1^	Monolayers, in vivo	Electron microscopy reported a significant destruction of MCCs in vitro and in vivo.	[34]
	Photofrin II	632.8	6	15 and 30 µg/mL	Beidegröm	Monolayers	Irradiated 15 μg/mL and 30 μg/mL of PS resulted in 71.9% and 90% apoptotic cell population, respectfully.	[35]
2nd	Ruthenium porphyrins	652	20	5 µM	ME300 ^2^	Monolayers	80% reduction in cell viability	[36]
	Halogenated porphyrin	630	10	10 µM	A375 ^3^	Monolayers	Improved singlet oxygen generation.	[37]
	Verteporfin	480	0.05–0.18	2 µg/mL	S91/13 ^4^	Monolayers	Significant cytodamage at a low concentration.	[38]
	m-THPC ^5^	514	10–25	10 µg/mL	B16 ^6^	Monolayers	PS showed an inhibitory effect in a dose and energy intensity dependent manner, overcoming apoptosis inhibitors.	[39]
	ZnPcOC ^7^	685	2.5–7.5	30 µM	Me45 8 ^8^	Monolayers	PDT triggered apoptosis in cancer with minimal effects on normal human cells.	[40]
	AlPcS4Cl ^9^ and Hyp ^10^	594 and 682	10	10 µM	A375	Monolayers	AlPcS4Cl inflicted more photodamage than Hyp, 15% and 10%, respectively.	[41]
	Ce6 ^11^	650	10	1.2 µM	B16	Monolayers	Ce6 and PDT resulted in 22.5% cell viability.	[42]
	Hyp	680	1	3 µM	A375, Mel-1 ^12^ and 501 Mel ^13^	Monolayers	Significant photodamage to mitochondria, endoplasmic reticulum, and cell membrane, which resulted in necroptosis.	[43]
	5-ALA ^14^	643	0.58	0.8 mM	A375	Monolayers	PDT caused loss of viability in a dose-dependent manner and elicited 90% apoptosis cell death in A375 cells.	[44]
	5-ALA and MPc ^15^	680	10	10 and 4 µM	A375	Monolayers	PDT reported a drastic reduction in cell viability ranging from 60% to 80% and induced apoptosis.	[45]
	Rhenium (I) complexes	330	528	5 µM	1205Lu ^16^	3-D cell cultures	Loss of spheroid integrity on the edges.	[46]
	Dinuclear Ruthenium(II) Complex	900	15.56	100 µM	C8161 ^17^	3-D cell cultures	Photodamage was observed in spheroid hypoxic regions.	[47]
	ZnPc	675 ± 15	340	20 µM	A375	3-D cell cultures	Significant photodamage was observed via induction of apoptosis.	[48]
	5-ALA	631	37	5 and 10 mM	B16F10 and B16G4F	In vivo	PDT noted a significant photodamage in both cell lines. Non-melanin pigmented B16G4F cells were more susceptible to the treatment than pigmented B16F10.	[49]
	5-ALA	420–1400	45–90	100 g/mL	Mel25 A375, B16-F0 and IH3T3	Monolayers, in vivo	Significant loss in cell viability was observed in vitro, whereas in vivo MT-rat mice tumors were unresponsive PDT.	[50]

^1^ Human malignant melanoma cells; ^2^ Human melanoma cells; ^3^ Human amelanotic melanoma; ^4^ The Cloudman S91/I3 mouse melanoma cell line; ^5^ Meta-tetrahydroxyphenylchlorin; ^6^ Murine melanoma cell line; ^7^ Zinc octacarboxyphthalocyanine; ^8^ Human pigmented malignant melanoma cells; ^9^ Aluminium (III) phthalocyanine chloride tetrasulphate; ^10^ Hypericin; ^11^ Chlorin e6; ^12^ Human melanoma cells; ^13^ Human melanoma cells; ^14^ 5-aminolevulinic acid; ^15^ Metallophthalocyanine; ^16^ Human metastatic melanoma cells; ^17^ Cutaneous melanoma cells.

**Table 2:** Various organic and inorganic NPs used for smart drug delivery in PDT and their benefits.

Type of NPs	NPs	Benefits	Ref.
Organic	Liposomes NPs	Biocompatible and biodegradable with minimal toxicity, can contain both hydrophilic and hydrophobic agents and protect encapsulated drugs from degradation by biological barriers.	[58]
	Micelle/polymeric NPs	High loading capacity, good biocompatibility, easy synthesis, versatile modification, and ability to evade biological barriers.	[60]
	Poly NPs (lactic-co-glycolic acid) (PLGA)	Superior nanocarriers due to their safety profile, no dark toxicity upon administration, and being biocompatible and biodegradable, and stable and poorly immunogenic.	[23]
	Dendrimers	Diverse functional surface molecules; flexible and tunable surfaces; highly monodispersed nanoconjugates; easy delivery of hydrophobic agents, hydrophilic internal cores, and multivalences; and biocompatible and fast clearance from body.	[61,62]
	Carbon nanotubes	High loading capacity, photothermal ablation, high permeability, highly modifiable surface, and good photodynamic properties.	[58,63]
Inorganic	Gold NPs (AuNP)	Exceptional stability, high surface to volume ratio, easy surface functionalization, high biocompatibility, high scattering energy, and strong absorption within the NIR region.	[64]
	Quantum dots	Tunable optical properties, excellent photo and chemical stability, high quantum yield, and size-tunable absorption bands.	[65]
	Silica NPs (inorganic)	Easy incorporation of both hydrophobic and hydrophilic drugs, efficient evasion from biological barriers, ease of functionalization, high biocompatibility, and high stability.	[63]
	Upconversion NPs	High optical absorption coefficients in the near NIR region and low phototoxicity.	[21]
	Ceramic NPs	High biocompatibility and stability, incorporation of both hydrophilic and hydrophobic molecules, and fast release of drugs.	[4,21]
	Magnetic NPs	Easy surface modification, selective photothermal destruction of cancer cells, strong superparamagnetic activity, and excellent PDT ability.	[5,66]

**Table 3:** Passive targeting PDT-PS nanocarrier systems in metastatic melanoma.

PS	Nanocarrier	Cell Line	Tumor Models	Outcome	Ref.
Aluminum chloride phthalocyanine (ClAlPc)	Solid lipid nanoparticles (SLN)	B16-F10	Monolayers	CIAIc-SLN decreased cell viability by 64.4%, while free PS showed a 54.1% decrease in B16F10 cells	[71]
Indocyanine green (ICG)	Chitosan-coated liposomes	B16-F10	Monolayers	ICG bioavailability increased by 2-fold in cells.	[72]
IR768 Daunorubicin (DRB)	polymeric micelles (PMs)	A375	Monolayers	Increased mitochondrial uptake, decreased cell viability below 20%.	[73]
Zinc Phthalocyanine Tetrasulphonate (ZnPcSO4)	poly (lactic acid-glycolic acid) (PLGA)	B16-F10	Monolayers	PS nanoconjugate induced 90% of cell death against 20% for free PS.	[74]
Protoporphyrin IX (PpIX)	poly (D, L lactic-co-glycolic acid) (PLGA)	B16-F10	Monolayers	PLGA maintained photophysical properties of PpIX, which reduced cell viability by 80%.	[75]
Zn-based porphyrin (Zn-EpPor)	Tobacco mosaic virus nanorods (TMVs)	B16-F10	Monolayers	PS-TMV exhibited improved cell uptake and stronger cytotoxicity than free PS.	[76]
5,10,15,20-Tetrakis(2,4-dihydroxyphenyl) porphyrin (POR)	Silver nanoparticles (AgNPs)	A375	Monolayers	PS-Ag showed in increased singlet oxygen quantum yield and cellular uptake than free PS.	[77]
Zinc monocarboxyphenoxy phthalocyanine (ZnMCPPc)	Gold nanoparticles (AuNPs)	A375	Monolayers	ZnMCPPc-Au showed a stronger PDT efficacy when compared to free ZnMCPPc.	[78]
Hypericin (Hyp)	1,2-dipalmitoyl-sn-glycero-3-phosphocholine (DPPC)	B16-F10	Monolayers	Hyp-DPPC showed an increased singlet oxygen quantum yield compared to free Hyp.	[79]
Verteporfin (Ver)	Mesoporous silica nanoparticles (MSNs)	B16-F10	Monolayers, in vivo 8-week-old female C57BL6/J mice	Ver-MSNs exhibited significant antiproliferative effects than free Ver and reduce tumor by 50.2 ± 6.6%.	[80]
Indocyanine green (ICG)	Hydrogen-peroxide-responsive protein biomimetic	B16-F10	Monolayers, in vivo 6–8-week-old BALB/c nude female mice	Improved stability, cellular uptake and phototoxicity	[81]
Palladium porphyrin (PdTCPP)	Layered double hydroxide (LDH)	B16-F10	Monolayers, in vivo 8-week-old male mice	PS-NP showed only 10% decrease in absorbance post PDT versus 85% loss by free PS, and decreased tumor growth by 7-fold in vivo.	[82]
Aluminum chloride phthalocyanine (ClAlPc)	Liposomes	WM1617	3-D cell cultures	PS-NP was efficiently taken up by 3-D tumor spheroids and induced more than 80% cell death.	[83]
Cabazitaxel (CTX)	psTKdC NAs	A375	In vivo, 6–8-week-old BALB/c nude female mice	Decreased tumor volume from 82.2 ± 41.4 mm^3^ to 21.5 ± 23.9 mm^3^ on day 0.	[84]
Zinc phthalocyanine (ZnPc)	Chitosan/methoxy polyethylene glycol-polylactic acid (CPP)	A431	In vivo, 6–8-week-old hairless female SKH-1 mice	PS-NP showed 75% cell death, compared to 50% for free PS.	[85]

**Table 4:** Metastatic melanoma active targeting delivery systems.

PS	Active PS Delivery System	Cell Line	Tumor Model	Outcomes	Ref.
Zinc phthalocyanine tetra-sulphonic acid (ZnPcS_4_)	Anti-Melanoma Inhibitory Activity (Anti-MIA) combined with AuNPs	A375	Monolayers	The bioconjugate concentrated the PS within the cytoplasm and nuclei, triggering a 65% apoptotic cell population	[91]
Ferrous chlorophyllin (Fe-CHL)	PLGA NPs loaded with cRGDyk peptide	B16-F10	Monolayers	The combination therapy showed enhanced accumulation of the PS and singlet oxygen generation in B16-F10 cells	[92]
Zinc ethynylphenyl porphyrin (Zn-EpPor)	Cowpea mosaic virus (CPMV) bioconjugated to dendron hybrids	B16-F10	Monolayers	2 PS-CPMV achieved a 2-fold increase in efficacy when compared to free PS.	[93]
Methylene blue (MB)	Naproxen amides (NAPs)	B16-F10	Monolayers	MB-NAP induced high levels of toxicity on MC-1 receptor-expressing B16-F10 cells, leaving only 4% of cells viable.	[94]
BODIPY (BDP)	Phenylthiourea (PTU)	B16-F10	Monolayers	BDP-PTU showed an enhanced cellular uptake, resulting in 20% cell viability.	[95]
Rose Bengal (RB)	Amphipathic peptide (AMP) C(KLAKLAK)2	B16-F10-Luc2	Monolayers, in vivo C57 mice	The target specificity and PDT effects of RB significantly reduced the viability of B16-F10-Luc2 cells to 6%.	[7]
Pyropheophorbide	Perfluorocarbons (PFCs) anchored onto hyaluronic acid (HA)	OM431	Monolayers, in vivo 4-week-old BALB/c male mice	The nanocomposite increased singlet oxygen production, which reduced cell viability to 30% in vitro and tumor weight to 0.05 g in vivo.	[56]
Indocyanine Green (ICG) With temozolomide (TMZ)	Hyaluronic acid (HA)-modified with Poly(amino-amine) (PAMAM)	A375	Monolayers, in vivo 6–8-week-old nude BALB/c female mice	ICG active nanophotosensitizer showed the strongest tumor cell-killing effect and revealed a cell viability of 17.1%.	[96]
IR820	Catalase (CAT) encapsulated in (PLGA) NPs	MV3	monolayers, in vivo 6–8-week-old BALB/c nude female mice	Displayed increased cellular uptake with 10% cell viability in vitro and a significant tumor regression in vivo.	[97]
Chlorin e6 (Ce6)	Anti-CD25	B16-F10	In vivo, C57BL/6-Tg (Foxp3-GFP) 90Pkraj/J mice	Ce6-CD25-targeted PDT induced apoptosis in 60–70% of melanoma tumors and caused tumor regression.	[98]

## Abbreviations

2D	Two-dimensional
3D	Three-dimensional
5-ALA	5-aminolevulinic acid
AMP	Amphipathic peptide
AgNPs	Silver nanoparticles
AlPcS4Cl	Aluminum (III) phthalocyanine chloride tetrasulphate
ATP	Adenosine triphosphate
AuNP	Gold nanoparticles
BCC	Basal cell carcinoma
Bcl-2	B-cell lymphoma 2
BDP	BODIPY
BRAF	B-Raf serine/threonine-protein
CAT	Catalase
Ce6	Chlorin e6
CPP	Chitosan/methoxy polyethylene glycol-polylactic acid
CPMV	Cowpea mosaic virus
CTLA-4	Cytotoxic T-lymphocyte-associated protein 4
CTX	Cabazitaxel
DPPC	1,2-dipalmitoyl-sn-glycero-3-phosphocholine
DRB	Daunorubicin
DTIC	Dacarbazine
ECM	Extracellular matrix
EPR	Enhanced permeability and retention effect
FDA	Food and drug administration
Fe-CHL	Ferrous chlorophyllin
HA	Hyaluronic acid
HpD	Hematoporphyrin derivative
Hyp	Hypericin
ICG	Indocyanine green
LDH	Layered double hydroxide
mAb	Monoclonal antibodies
MB	Methylene blue
MC1	Melanocortin receptor
MIA	Melanoma inhibitory activity
MPc	Metallophthalocyanine
m-THPC	Meta-tetrahydroxyphenylchlorin
MM	Metastatic melanoma
MSNs	Mesoporous silica nanoparticles
NMSC	Non-melanoma skin cancer
NAP	Naproxen amides
NP	Nanoparticle
PEG	Polyethylene glycol
PAMAM	Poly amino-amine
PD-1	Programmed cell death 1
PDT	Photodynamic therapy
PdTCPP	Palladium porphyrin
PFCs	Perfluorocarbons
PMs	polymeric micelles
Pcs	phthalocyanines
PLGA	Poly lactic-co-glycolic acid
POR	5,10,15,20-Tetrakis(2,4-dihydroxyphenyl) porphyrin
PpIX	Protoporphyrin IX
PS	Photosensitizer
PTU	Phenylthiourea
RB	Rose Bengal
RGD	Arginylglycylaspartic acid
ROS	Reactive oxygen species (ROS),
SCC	Squamous cell carcinoma
TMV	Tobacco mosaic virus nanorods
TMZ	Temozolomide
TRAIL	TNF-related apoptosis inducing ligand
Ver	Verteporfin
WHO	World Health Organization
UV	Ultraviolet radiation
Zn-EpPor	Zinc n-based porphyrin
ZnMCPPc	Zinc monocarboxyphenoxy phthalocyanine
ZnPcOC	Zinc octacarboxyphthalocyanine
ZnPcS	Zinc phthalocyanine
ZnPcSO4	Zinc Phthalocyanine Tetrasulphonate
